# COVID-19: Training activities, adherence, and use of personal protective equipment in Primary Health Care

**DOI:** 10.1590/0034-7167-2023-0179

**Published:** 2025-01-10

**Authors:** Kelly Aline Rodrigues Costa, Fernanda Moura Lanza, Francisco Carlos Félix Lana, Camila Custódio da Silva, Camila Cristina Gregório de Assis, Cosme Rezende Laurindo, Herica Silva Dutra, Angélica da Conceição Oliveira Coelho

**Affiliations:** I Universidade Federal de São João del-Rei. Divinópolis, Minas Gerais, Brazil; II Universidade Federal de Minas Gerais. Belo Horizonte, Minas Gerais, Brazil; III Universidade Federal de Juiz de Fora. Juiz de Fora, Minas Gerais, Brazil

**Keywords:** COVID-19, Primary Health Care, Health Personnel, Personal Protective Equipment, Training Activities, COVID-19, Atención Primaria de Salud, Personal de Salud, Equipo de Protección Personal, Actividades Formativas

## Abstract

**Objective:**

to analyze the association between participation in training activities and the adherence to and use of personal protective equipment by workers and professionals involved in Health Residency Programs in Primary Health Care during the COVID-19 pandemic.

**Methods:**

a cross-sectional study in Brazil between August/2020 and March/2021. We utilized the EPI-APS COVID-19 instrument and its adapted version for resident professionals.

**Results:**

455 PHC workers and 102 residents participated in the study. Among them, 54.5% and 55.9%, respectively, engaged in training activities. We observed an association between participation in training activities and the proper use of gloves (p<0.001), gowns (p=0.009), goggles/face shields (p=0.002), and overall adherence (p<0.001) among PHC workers, and the proper use of surgical masks (p=0.028) among residents. Adherence rates of ≥75% were identified in 6.9% of PHC workers and none among the residents.

**Conclusion:**

training activities are associated with increased adherence to and proper use of PPE.

## RESUMEN

**Objetivo:**

analizar la relación de la realización de actividades formativas con adhesión y uso de los equipos de protección personal por los trabajadores y profesionales vinculados a Programas de Residencia Hospitalaria en la Atención Primaria de Salud en la pandemia de COVID-19.

**Métodos:**

estudio transversal, desarrollado en Brasil, entre agosto/2020 y marzo/2021. Utilizado el instrumento EPI-APS COVID-19 y su versión adaptada para profesionales residentes.

**Resultados:**

participaron 455 trabajadores de la Atención Primaria y 102 residentes. De estos, 54,5% y 55,9%, respectivamente, realizaron actividades de capacitación. Hubo relación entre realización de actividades formativas y uso adecuado de guantes (p<0,001), delantal/bata clínica (p=0,009), gafas de seguridad/protección facial (p=0,002) y adhesión (p<0,001) entre los trabajadores de la Atención Primaria; y uso adecuado de cubrebocas quirúrgicos (p=0,028) entre residentes. Identificado adhesión ≥75% por 6,9% de los trabajadores de la Atención Primaria y por ningún residente. **Conclusión:** actividades formativas tienen relación con adhesión y uso adecuado de equipos de protección personal.

## INTRODUCTION

In addressing the COVID-19 pandemic in Brazil, Primary Health Care (PHC) professionals have played a critical role in mapping, monitoring, identifying, and managing suspected and/or confirmed COVID-19 cases with mild symptoms, identifying more severe cases requiring specialized care, and monitoring convalescent cases^(1)^. In this context, a subset of healthcare professionals also performs essential activities in combating COVID-19 in Brazil: those affiliated with Health Residency Programs. These programs represent postgraduate specialization primarily conducted through practical activities (80%) ^(2)^, aimed at qualifying professionals committed to the principles and guidelines of the Unified Health System (SUS)^(3)^.

Protecting healthcare workers is essential for preventing COVID-19 transmission in the workplace^(4,5)^ since they are exposed to high viral loads and often work under inadequate conditions^(4,6)^. Measures have been reinforced, such as simple hand hygiene with soap and water or antiseptic hand rubs, physical distancing to avoid crowding, cleaning and disinfection of environments and surfaces, ensuring good ventilation, respiratory etiquette, and the use of personal protective equipment (PPE) in addition to those already used in daily practices^(4)^. Using N95/PFF2 masks and face shields has become part of the routine for PHC professionals during aerosol-generating procedures^(5)^.

To benefit from the protection offered by PPE, workers must select it appropriately and correctly perform donning, doffing, and disposal^(7,8)^. However, evidence indicates a lack of professional confidence regarding the proper use and correct donning and doffing procedures^(9,10,11)^. Therefore, conducting training activities is essential to encourage the appropriate and effective use of PPE^(9,12,13, 14)^, providing effective and safe preventive measures for healthcare workers, patients, and the entire healthcare team^(9)^.

Several aspects justify this investigation, including: 1) the impact of the COVID-19 pandemic on the daily lives of healthcare workers, their environment, and work demands^(1)^; 2) the recommendation of PPE use as the primary preventive measure to ensure workers’ protection against viral contamination^(6)^; 3) the importance of proper PPE use by healthcare professionals in the pandemic context to avoid healthcare-associated infections (HAIs)^(4,5)^; and 4) evidence that training activities can equip healthcare professionals with the knowledge to perform their tasks with greater confidence and safety^(15,16,17)^. Therefore, this study hypothesizes an association between participation in training activities and adherence to and proper use of PPE by PHC workers and professionals affiliated with Health Residency Programs during the COVID-19 pandemic.

## OBJECTIVE

To analyze the association between participation in training activities and PPE adherence to and use by workers and professionals involved in Health Residency Programs in PHC during the COVID-19 pandemic.

## METHODS

### Ethical aspects

This study followed national and international ethical guidelines. The Human Research Ethics Committee approved it under the Certificate of Ethical Appreciation number 30933220.7.0000.5147, in compliance with Resolution 466/12. We obtained Informed Consent from all research participants through their recorded agreement in a form made available on the free KoboToolbox platform.

### Study design, period, and location

We conducted a cross-sectional, descriptive, and analytical study in Brazil between August 2020 and March 2021. This study is part of the research project “Use of Personal Protective Equipment by Health Professionals in the Fight against COVID-19 - EPI COVID-19 Brazil.” We guided our work by the EQUATOR Network’s Strengthening the Reporting of Observational Studies in Epidemiology (STROBE) guidelines and the Checklist for Reporting Results of Internet E-Surveys (CHERRIES).

### Population, inclusion criteria, and sample

We invited all PHC workers listed in the National Register of Health Establishments (CNES) to participate in the study, including nurses, physicians, physiotherapists, speech therapists, dentists, nutritionists, pharmacists, psychologists, social workers, nursing technicians, nursing assistants, community health agents, oral health agents, oral health technicians, administrative-technical assistants, and receptionists (723,310 professionals). Additionally, we invited professionals involved in Health Residency Programs who work in PHC, including physicians, nurses, physiotherapists, dentists, speech therapists, social workers, psychologists, nutritionists, and pharmacists (4,716 professionals). All participants met the criterion of performing essential work activities in the fight against COVID-19, meaning they had daily and direct contact with the virus^(1,18)^. This study employed a convenience sample of those who voluntarily agreed to participate during the data collection period.

### Study protocol

We collected data in a virtual environment using the free KoboToolbox platform. To reduce selection bias in this online research, we utilized various dissemination methods for the “EPI COVID-19 Brazil” study^(19)^. Participants accessed the questionnaire after agreeing to the Informed Consent Form (ICF)^(19)^.

For data collection from PHC professionals, we used the validated EPI APS COVID-19 instrument^(20)^, which consists of 31 items distributed across eight domains: 1) Disposable cap or hood; 2) Gloves; 3) Safety behavior; 4) N95 mask; 5) Hand hygiene; 6) Disposable gown or apron; 7) Disposable surgical mask; 8) Goggles or face shield. We applied an adapted version of the instrument for professionals involved in Health Residency Programs, replacing the term “PHC service” with “health service” in nine questions and excluding the term “PHC” from one question^(21)^. Our research team developed questions to characterize participants regarding sociodemographic aspects, professional training, and the workplace.

The dependent variables in this study were the proper use of PPE and adherence to PPE protocols. Independent variables included being a PHC professional or affiliated with Health Residency Programs, age, participation in training activities focused on PPE use, and the types of training activities conducted.

### Data analysis and statistics

We exported the data collected on the KoboToolbox platform to a Microsoft Office Excel spreadsheet for consistency evaluation and database organization. We then exported the data to the Statistical Package for the Social Sciences (SPSS) software, version 21.0, for statistical analysis. We first conducted the Kolmogorov-Smirnov normality test. Descriptive statistical analysis (absolute and relative frequency, mean, and standard deviation) was used to characterize the sample. To analyze adherence to PPE use among healthcare workers, we employed measures of central tendency (mean, median) and dispersion (standard deviation and interquartile range).

To assess proper PPE use, we considered a professional to be using PPE correctly when they scored full points in each domain (items related to the absence of PPE were not included in this analysis). Adherence to PPE use was measured by calculating the individual score (number of domains with proper use / total number of domains answered × 100). The adherence score adopted for PPE use was 75% or higher, consistent with other studies in the literature^(22,23)^. Thus, adherence indicates the extent to which PHC workers and professionals affiliated with Health Residency Programs follow proper PPE use to protect themselves against COVID-19.

We used chi-square or Fisher’s exact tests to analyze the association between dependent and independent variables, adopting a significance level of p ≤ 0.05. Prevalence estimates related to participation in training activities and proper PPE use were calculated with a 95% confidence interval (95% CI). We analyzed the relationship between adherence and participation in PPE training activities among healthcare workers and residents using the Mann-Whitney test.

## RESULTS

We included 557 healthcare workers in the study, with 455 (81.7%) from PHC and 102 (18.3%) affiliated with Health Residency Programs. The average age was 37.3 years (standard deviation - SD±8.9) for PHC workers and 28.3 years (SD±6.7) for those in Residency Programs. The majority of participants were between 25 and 39 years old (344; 61.8%), identified as cisgender women (449; 80.6%), had a partner (279; 50.1%), and resided in the Southeast region of Brazil (376; 67.5%).

Regarding professional data, 443 (79.5%) held a higher education degree, with most (281; 63.4%) reporting specialization in health. The predominant professional category was nurses, with 235 participants (42.2%). The average length of service in PHC was 9.6 years (SD±7.7), while for those in Residency Programs, it was 13.8 months (SD±9.5).

When asked about their knowledge and techniques related to the recommended PPE for their healthcare services, 278 (49.9%) partially agreed with the statement. The majority (454; 81.5%) fully agreed on the need to conduct training activities related to PPE use.

Table 1 presents the characterization related to PPE training activities. We observed that 305 (54.8%) participants reported having participated in training activities during the pandemic.

**Table 1:** Characterization of personal protective equipment training activities among Primary Health Care workers and professionals affiliated with Health Residency Programs. Brazil, 2021 (N = 557)

Variables		Participant’s professional affiliation	
		PHC n (%)	Residency n (%)
Participation in training activities	Yes	248 (54.5)	57 (55.9)
	No	207 (45.5)	45 (44.1)
Modality			
Online / Distance learning	Yes	188 (75.8)	48 (84.2)
	No	60 (24.2)	9 (15.8)
In-person	Yes	76 (30.6)	21 (36.8)
	No	172 (69.4)	36 (63.2)
Hybrid	Yes	10 (4.0)	1 (1.8)
	No	238 (96.0)	56 (98.2)
Duration*			
1 to 4 hours		86 (34.7)	16 (28.1)
5 to 10 hours		52 (21.0)	9 (15.8)
11 to 30 hours		51 (20.6)	21 (36.8)
More than 31 hours		58 (23.4)	11 (19.3)
Not specified		1 (0.4)	0 (0.0)

* Categorization of duration based on percentiles.

We found an association between participation in PPE training activities and the proper use of gloves (p < 0.001), gowns (p = 0.009), and goggles/face shields (p = 0.002) among PHC workers. The prevalence of proper PPE use among PHC workers who participated in training activities was 1.56 times higher for gloves, 1.54 times higher for gowns, and 1.37 times higher for goggles/face shields than those who did not participate in training activities (Table 2).

**Table 2:** Association between participation in training activities and proper personal protective equipment use, safety behavior, and hand hygiene among Primary Health Care workers. Brazil, 2021 (n = 455)

**Training activities**	**Proper PPE use n (%)**							
	**Cap**		**Gloves**		**Gown/Apron**		**Goggles/Face shield**	
	**Yes**	**No**	**Yes**	**No**	**Yes**	**No**	**Yes**	**No**
Yes	26 (15.2)	145 (84.8)	83 (48.5)	88 (51.5)	74 (48.7)	78 (51.3)	114 (71.2)	46 (28.8)
No	13 (10.1)	116 (89.9)	40 (31.0)	89 (69.0)	29 (31.5)	63 (68.5)	52 (52.0)	48 (48.0)
*p* value	0.191^*^		<0.001^*^		0.009^*^		0.002^*^	
PR (95% CI)		1.50 (0.807-2.820)		1.56 (1.159-2.113)		1.54 (1.096-2.175)		1.37 (1.108-1.695)	
**Training activities**	**Proper PPE use n (%)**				**Safety behavior n (%)**		**Hand hygiene n (%)**	
	**Surgical mask**		**N95 mask**					
	**Yes**	**No**	**Yes**	**No**	**Yes**	**No**	**Yes**	**No**
Yes	52 (24.8)	158 (75.2)	79 (59.4)	54 (40.6)	35 (14.1)	213 (85.9)	132 (53.2)	116 (46.8)
No	48 (27.4)	127 (72.6)	40 (46.0)	47 (54.0)	20 (9.7)	187 (90.3)	92 (44.4)	115 (55.6)
*p* value	0.552^*^		0.051^*^		0.147^*^		0.062^*^	
PR (95% CI)		0.90 (0.644-1.265)		1.29 (0.989-1.688)		1.46 (0.871-2.451)		1.19 (0.989-1.451)	

PPE – Personal Protective Equipment; n – absolute frequency; PR – prevalence ratio; 95% CI – 95% confidence interval; * chi-square test.

In analyzing the association between participation in training activities and proper PPE use among professionals affiliated with Health Residency Programs, we obtained statistically significant values for the proper use of surgical masks (p = 0.028). This usage was 1.42 times higher among residents who participated in training activities than those who did not (Table 3).

**Table 3:** Association between participation in training activities and proper personal protective equipment use, safety behavior, and hand hygiene among professionals affiliated with Health Residency Programs. Brazil, 2021 (n = 102)

**Training activities**	**Proper PPE use n (%)**									
	**Cap**		**Gloves**		**Gown/Apron**		**Goggles/Face shield**	
	**Yes**	**No**	**Yes**	**No**	**Yes**	**No**	**Yes**	**No**
Yes	9 (23.7)	29 (76.3)	0 (0.0)	43 (100.0)	9 (26.5)	25 (73.5)	22 (57.9)	16 (42.1)
No	4 (17.4)	19 (82.6)	3 (11.1)	24 (88.9)	8 (40.0)	12 (60.0)	6 (42.9)	8 (57.1)
*p* value	0.749€		0.053€		0.301*		0.335*	
PR (95% CI)	1.36 (0.473-3.922)		------		0.66 (0.305-1.438)		1.351 (0.696-2.621)	
**Training activities**	**Proper PPE use n (%)**				**Safety behavior n (%)**		**Hand hygiene n (%)**	
	**Surgical mask**		**N95 mask**					
	**Yes**	**No**	**Yes**	**No**	**Yes**	**No**	**Yes**	**No**
Yes	36 (75.0)	12 (25.0)	19 (52.8)	17 (47.2)	1 (1.7)	59 (98.3)	21 (35.0)	39 (65.0)
No	21 (52.5)	19 (47.5)	8 (44.4)	10 (55.6)	0 (0.0)	42 (100.0)	13 (31.0)	29 (69.0)
*p* value	0.028*		0.564*		1.000€		0.670*	
PR (95% CI)	1.42 (1.020-2.001)		1.18 (0.650-2.168)		------		1.13 (0.641-1.996)	

PPE – Personal Protective Equipment; n – absolute frequency; PR – prevalence ratio; 95% CI – 95% confidence interval; * chi-square test; € Fisher’s exact test.

On the one hand, when evaluating whether professional affiliation was associated with proper PPE use, safety behavior, and hand hygiene (Table 4), we observed a statistically significant difference, with the PHC group showing a 3.28 times higher prevalence of proper glove use (p < 0.001) and a 1.35 times higher rate of hand hygiene (p = 0.018). On the other hand, surgical mask use was 47% lower (p < 0.001) among PHC professionals than those in Residency Programs.

**Table 4:** Association between professional affiliation and proper personal protective equipment use, safety behavior, and hand hygiene. Brazil, 2021 (N = 557)

**Professional affiliation**	**Proper PPE use n (%)**							
	**Cap**		**Gloves**		**Gown/Apron**		**Goggles/Face shield**	
	**Yes**	**No**	**Yes**	**No**	**Yes**	**No**	**Yes**	**No**
PHC	39 (13.0)	261 (87.0)	123 (41)	177 (59.0)	10 (42.2)	141 (57.8)	166 (63.8)	94 (36.2)
Residency	7 (11.5)	54 (88.5)	8 (12.5)	56 (87.5)	21 (43.8)	27 (56.2)	28 (57.1)	21 (42.9)
*p* value	0.745*		<0.001*		0.844*		0.373*	
PR (95% CI)	1.13 (0.532-2.413		3.28 (1.691-6.360)		0.96 (0.678-1.373)		1.11 (0.862-1.448)	
**Professional affiliation**	**Proper PPE use n (%)**				**Safety behavior n (%)**		**Hand hygiene n (%)**	
	**Surgical mask**		**N95 mask**					
	**Yes**	**No**	**Yes**	**No**	**Yes**	**No**	**Yes**	**No**
PHC	100 (26.0)	285 (74.0)	119 (54.1)	101 (45.9)	55 (12.1)	400 (87.9)	224 (49.2)	231 (50.8)
Residency	43 (49.4)	44 (50.6)	31 (49.2)	32 (50.8)	0 (0.0)	102 (100.0)	37 (36.3)	65 (63.7)
*p* value	<0.001*		0.493*		<0.001€		0.018*	
PR (95% CI)	0.526 (0.401-0.689)		1.09 (0.832-1.453)		------		1.35 (1.032-1.784)	

PPE – Personal Protective Equipment; PHC - Primary Health Care; n – absolute frequency; PR – prevalence ratio; 95% CI – 95% confidence interval; * chi-square test; € Fisher’s exact test.

Among the barriers to proper PPE use in healthcare services (data not shown in tables), participants mentioned the absence of training activities (291; 52.2%), lack of knowledge (227; 40.8%), lack of infrastructure (224; 40.2%), and PPE shortages (310; 55.7%).

Regarding adherence to proper PPE use by PHC workers (Table 5), we observed that 17 (3.7%) had 75% adherence, 14 (3%) showed adherence rates between 76% and 99%, and only 1 (0.2%) demonstrated 100% adherence to PPE use. No participant in the residents’ group showed adherence to PPE use equal to or greater than 75% (Table 5).

**Table 5:** Adherence to proper personal protective equipment use among Primary Health Care workers and professionals affiliated with Health Residency Programs. Brazil, 2021 (N = 557)

Professional affiliation	Minimum	1st Quartile	Mean (SD)	Median	3rd Quartile	Maximum
PHC (%)	0.0	12.5	33.3 (±25.0)	33.3	50.0	100.0
Residents (%)	0.0	16.2	28.4 (±19.0)	29.0	43.0	67.0

*SD – standard deviation; PHC - Primary Health Care.*

In the analysis of the relationship between adherence and participation in PPE training activities by healthcare workers, we observed that such training activities significantly impacted PPE adherence (p < 0.001).

## DISCUSSION

This study evaluated the association between participation in training activities and the adherence to and use of PPE among PHC workers and professionals affiliated with Health Residency Programs working in PHC services. Our findings showed that participation in training activities is associated with the proper use of gloves, gowns, and goggles/face shields among PHC workers, and with the proper use of surgical masks among residents. These activities positively affected adherence to PPE use in both groups.

Other studies have also associated training activities with the proper use of PPE^(1,24)^. Researchers have demonstrated that knowledge and understanding of biosafety principles, as well as the correct handling of PPE during use, donning, and doffing, lead to patient and professional safety and, consequently, to the minimization of COVID-19 contamination^(7,8,23)^. To achieve this, it is essential to adopt infection control protocols and provide PPE such as masks, gowns, goggles, face shields, and gloves^(5)^.

However, participants in this study exhibited low adherence to PPE use, consistent with findings from other investigations^(9,25,26)^. This result suggests a possible undervaluation of existing risks in the workplace by professionals^(27)^. Various factors influence adherence to PPE as a preventive measure against COVID-19: 1) organizational factors, such as safety climate, guidelines, and availability of training programs^(13,22,28)^; 2) environmental factors, including the physical environment and PPE availability^(13,22,29)^; and 3) individual factors, such as knowledge^(11)^ and positive attitudes toward PPE selection and use, and greater clinical experience^(22,29)^.

Regarding the proper use of PPE by PHC workers, the item “goggles/face shield” showed the highest percentage of proper use. This evidence may be justified by the importance of this PPE for protecting workers during activities involving exposure to bodily secretions^(4,30)^. Similarly, an online survey conducted in Qatar with 757 PHC professionals reported that 55.4% of workers used goggles/face shields during encounters with suspected or confirmed COVID-19 cases^(30)^. In Brazil, a study with 106 oral health professionals showed proper adherence to aerosol precaution measures, notably face shields (75.5%), N95 masks (62.3%), and gowns (53.8%)^(31)^.

Regarding surgical masks, a previous study reported a 90.6% rate of proper use during encounters with suspected or confirmed COVID-19 cases among PHC workers^(30)^. However, in the present study, proper use was more prevalent among resident professionals, which could be attributed to the fact that residents, in training, are frequently evaluated by their teams and preceptors regarding behaviors adopted to ensure protection against COVID-19.

Studies conducted during the COVID-19 pandemic have shown that gloves were among the most commonly used PPE^(30,31,32)^. In this study, PHC workers were 3.28 times more likely to use gloves properly than resident professionals. The use of caps was identified as the most challenging PPE for participants to use correctly, corroborating findings from a pre-pandemic study with hospital nursing professionals^(33)^. However, another study showed that oral health professionals reported caps as the most frequently used PPE during the pandemic^(31)^.

In this study, the main barriers reported by healthcare workers to proper PPE use were the absence of training activities and lack of knowledge about control and prevention measures. However, this issue extends beyond COVID-19. A recent study in Colorado’s PHC and emergency services found that only 23% of healthcare professionals exposed to monkeypox used all recommended PPE (mask, gloves, gowns, goggles, or face shield)^(34)^. This low adherence could be explained by a lack of knowledge about the disease’s clinical manifestations, community transmission, and PPE recommendations^(35)^. Given that frontline healthcare professionals combating COVID-19 are more likely to come into contact with the virus, it is essential to provide them with training activities to adopt conscious and safe measures in their work environments^(34)^.

The “safety behavior” domain, assessed by the data collection instrument used in this study, showed the worst results among both categories of participating professionals. This finding aligns with a Brazilian study demonstrating that not all biosafety precautions are implemented in daily work activities^(31)^. Additionally, Additionally, adherence to practices such as hand hygiene^(25)^ and environmental decontamination^(36)^ is necessary alongside the proper use of PPE for safely performing work activities.

Proper hand hygiene was reported by 49.2% of PHC workers and 36.3% of residents. In Ethiopia, 56.7% of workers practiced good hand hygiene during the COVID-19 pandemic^(25)^. However, a study conducted with PHC workers in Brazil before the COVID-19 pandemic highlighted that barriers to adopting standard precautionary measures included the perception that infection risks in PHC are lower than in hospital settings and weaknesses in the availability of training^(37)^.

Given the pandemic scenario, the importance of preventive measures such as social distancing, and the need for continuous professional development, PHC workers and residents participated in online/distance learning training activities on quality preventive measures in their workplaces. These training activities were offered in an online/theoretical format with reduced hours due to the urgency of providing healthcare professionals with relevant and highly demanded information to combat COVID-19^(16,17)^.

The literature has shown that healthcare professionals in various countries (such as Australia^(15)^, Brazil^(26)^, China^(38)^, and Ethiopia^(39)^) received insufficient training to face COVID-19. In this context, the role of authorities^(40)^ in providing online and free training to professionals working during the pandemic is crucial, aiming to equip them with the necessary scientific knowledge to perform their activities with the utmost safety^(19,26)^. As a form of knowledge translation, researchers in this study developed the course “Biosafety: Best Practices for Working during COVID-19,” offered virtually and free to all study participants. The course was structured into three modules, with 15 hours of instruction and certification^(19)^.

Thus, investing in resources and educational activities for these workers significantly impacts on the quality of care, as it helps prevent healthcare-associated infections (HAIs) such as COVID-19^(25)^. In this sense, qualified professionals provide greater safety to their teams and, consequently, more confidence to face the pandemic^(7,15)^.

### Study limitations

One limitation of this study is its cross-sectional design, which must be interpreted considering its inherent characteristics. Additionally, we conducted the research in a virtual environment, which can introduce selection bias^(41)^, reflecting a sample of participants who were influenced by the strategies used to disseminate the study and who voluntarily chose to participate.

### Contributions to the field

This study reveals concerning data on the proper use of PPE and adherence to its use during the COVID-19 pandemic within a sample of healthcare workers and residents in Brazilian PHC services (42.2% with higher education in nursing). These results suggest potential gaps in academic and in-service training concerning biosafety measures. We recommend incorporating the topic of “biosafety” throughout the educational process to consolidate knowledge for consistent application in practice. The aim is to ensure the safety of workers and users, as well as to improve the quality of care provided, thereby preventing the transmission of HAIs in the workplace, not only in the context of COVID-19 but also for other infectious diseases.

## CONCLUSIONS

We conclude that participation in training activities is associated with adherence to PPE use and the proper use of surgical masks among resident professionals during the COVID-19 pandemic. For PHC workers, it is associated with using gloves, gowns, and goggles/face shields. Resident professionals demonstrated adherence below the reference value used in this study, while only 6.9% of PHC workers achieved adherence rates equal to or greater than 75%.

We suggest conducting future studies to identify the facilitators and barriers to adherence and proper PPE use in PHC services, including in the current post-pandemic period.
