# Supplementary material for: COVID-19: Training activities, adherence, and use of personal protective equipment in Primary Health Care
Source: Rev Bras Enferm. 2025 Jan 10;77(Suppl 1):e20230179. doi: 10.1590/0034-7167-2023-0179 (PMC11726901; doi:10.1590/0034-7167-2023-0179)
Supplement: Translation

# Translation: COVID-19: Atividades de capacitação, adesão e uso de equipamentos de proteção individual na Atenção Primária

**DOI:** 10.1590/0034-7167-2023-0179pt

**Authors:** Kelly Aline Rodrigues Costa, Fernanda Moura Lanza, Francisco Carlos Félix Lana, Camila Custódio da Silva, Camila Cristina Gregório de Assis, Cosme Rezende Laurindo, Herica Silva Dutra, Angélica da Conceição Oliveira Coelho

**Affiliations:** I Universidade Federal de São João del-Rei. Divinópolis, Minas Gerais, Brasil; II Universidade Federal de Minas Gerais. Belo Horizonte, Minas Gerais, Brasil; III Universidade Federal de Juiz de Fora. Juiz de Fora, Minas Gerais, Brasil

**Keywords:** COVID-19, Atenção Primária à Saúde, Profissionais da Saúde, Equipamentos de Proteção Individual, Atividades de Capacitação

## Abstract

**Objetivo:**

analisar a associação da realização de atividades de capacitação com adesão e uso dos equipamentos de proteção individual pelos trabalhadores e profissionais vinculados a Programas de Residência em Saúde na Atenção Primária à Saúde na pandemia de COVID-19.

**Métodos:**

estudo transversal, desenvolvido no Brasil, entre agosto/2020 e março/2021. Utilizou-se o instrumento EPI-APS COVID-19 e sua versão adaptada para profissionais residentes.

**Resultados:**

participaram 455 trabalhadores da Atenção Primária e 102 residentes. Destes, 54,5% e 55,9%, respectivamente, realizaram atividades de capacitação. Houve associação entre realização de atividades de capacitação e uso adequado de luvas (p<0,001), avental/ capote (p=0,009), óculos/proteção facial (p=0,002) e adesão (p<0,001) entre os trabalhadores da Atenção Primária; e uso adequado de máscara cirúrgica (p=0,028) entre residentes. Identificou-se adesão ≥75% por 6,9% dos trabalhadores da Atenção Primária e por nenhum residente.

**Conclusão:**

atividades de capacitação têm associação com adesão e uso adequado de equipamentos de proteção individual.

## INTRODUÇÃO

No enfrentamento da pandemia de COVID-19 no Brasil, os profissionais das equipes de Atenção Primária à Saúde (APS) desempenham importante papel no mapeamento, monitoramento, identificação e condução dos casos suspeitos e/ou confirmados de COVID-19 com sintomas leves, na identificação de casos mais graves que necessitam de atenção especializada e no acompanhamento dos casos convalescentes^(1)^. Nesse contexto, um subconjunto de profissionais de saúde também desenvolve atividades essenciais para o enfrentamento da COVID-19 no Brasil: os vinculados a Programas de Residência em Saúde. Trata-se de pós-graduação latu sensu desenvolvida predominantemente por meio de atividades práticas (80%)^(2)^ para qualificar profissionais comprometidos com os princípios e diretrizes do SUS^(3)^.

Assim, assegurar a proteção dos trabalhadores de saúde é fundamental para prevenir a transmissão da COVID-19 no ambiente de trabalho^(4,5)^ já que estão expostos a alta carga viral e submetidos a condições de trabalho, frequentemente, inadequadas^(4,6)^. Para isso, medidas foram reforçadas, tais como as de higienização simples das mãos com água e sabão ou com fricção antisséptica das mãos com produto alcóolico, distanciamento físico entre as pessoas evitando-se aglomerações, limpeza e desinfecção de ambientes e superfícies, ambientes com boa ventilação, etiqueta respiratória e uso de equipamentos de proteção individual (EPI) além dos já utilizados em suas práticas diárias^(4)^. Mostra-se que o uso de máscara N95/PFF2 e do protetor facial (*face shield*) passaram a fazer parte da rotina de trabalho dos profissionais de saúde da APS (Atenção Primária à Saúde) durante a realização de procedimentos geradores de aerossóis^(5)^.

Para desfrutarem da proteção oferecida pelos EPIs, os trabalhadores devem selecioná-los adequadamente e realizar a correta paramentação, desparamentação e descarte^(7,8)^. No entanto, há evidências de insegurança profissional quanto ao uso adequado e à correta paramentação e desparamentação^(9,10,11)^. Por isso, realizar atividades de capacitação é essencial para incentivar o uso adequado e efetivo dos EPIs^(9,12,13,14)^, o que proporcionará medidas preventivas eficazes e seguras aos trabalhadores da saúde, aos pacientes e a toda equipe de trabalho^(9)^.

Alguns aspectos justificam a realização desta investigação, tais como: i) o impacto da pandemia de COVID-19 no cotidiano dos trabalhadores da saúde, em seu ambiente e suas demandas laborais^(1)^; ii) a recomendação do uso de EPIs como principal medida preventiva para assegurar a proteção dos trabalhadores contra a contaminação pelo vírus^(6)^; iii) a importância do uso adequado de EPIs pelos profissionais de saúde em face do contexto pandêmico [ou não] para evitar as infecções relacionadas a assistência à saúde (IRAS)^(4,5)^; e iv) as evidências de que atividades de capacitação podem proporcionar aos profissionais de saúde conhecimento para executar suas tarefas laborais com maior confiança e segurança^(15,16,17)^. Diante disso, a hipótese deste estudo foi de que há associação entre a realização de atividades de capacitação e a adesão e uso adequado dos EPIs pelos trabalhadores da APS e por profissionais vinculados a Programas de Residência em Saúde na pandemia de COVID-19.

## OBJETIVO

Analisar a associação entre a realização de atividades de capacitação e a adesão e o uso dos EPIs pelos trabalhadores e profissionais vinculados a Programas de Residência em Saúde na APS na pandemia de COVID-19.

## MÉTODOS

### Aspectos éticos

O estudo foi conduzido de acordo com as diretrizes de ética nacionais e internacionais e foi aprovado pelo Comitê de Ética em Pesquisa com Seres Humanos, com Certificado de Apresentação de Apreciação Ética de nº 30933220.7.0000.5147, em conformidade com a Resolução 466/12. O Consentimento Livre e Esclarecido foi obtido de todos os participantes da pesquisa por meio de registro de anuência em termo disponibilizado na plataforma gratuita KoboToolbox.

### Desenho, período e local do estudo

Estudo transversal, descritivo e analítico realizado no Brasil, entre agosto de 2020 e março de 2021. Este estudo faz parte da pesquisa “Uso de equipamentos de proteção individual pelos profissionais de saúde no combate à COVID-19 - EPI COVID-19 Brasil”. Foi norteado pelos guias da Rede EQUATOR *Strengthening the Reporting of Observational Studies in Epidemiology* (STROBE) e *Checklist for Reporting Results of Internet E-Surveys* (CHERRIES).

### População, critérios de inclusão e amostra

Foram convidados a participar do estudo todos os trabalhadores da APS listados no Conselho Nacional de Estabelecimentos em Saúde (CNES): enfermeiro, médico, fisioterapeuta, fonoaudiólogo, cirurgião dentista, nutricionista, farmacêutico, psicólogo, assistente social, técnico de enfermagem, auxiliar de enfermagem, agente comunitário, agente de saúde bucal, técnico em saúde bucal, assistente técnico administrativo e recepcionista (723.310 profissionais). Além destes, foram convidados os profissionais vinculados aos Programas de Residência em Saúde que atuam na APS: médico, enfermeiro, fisioterapeuta, cirurgião dentista, fonoaudiólogo, assistente social, psicólogo, nutricionista e farmacêutico (4.716 profissionais). Todos preenchiam o critério de desempenhar atividades laborais indispensáveis ao enfrentamento da COVID-19, ou seja, tinham contato diário e direto com o vírus^(1,18)^. Sendo assim, trata-se de uma amostra por conveniência, isto é, composta por aqueles que concordaram em participar voluntariamente da pesquisa durante o período de coleta de dados.

### Protocolo do estudo

A coleta de dados ocorreu em ambiente virtual pela plataforma gratuita KoboToolbox. A utilização de diferentes meios de divulgação da pesquisa “EPI COVID-19 Brasil”^(19)^ foi a estratégia empregada para reduzir o viés de seleção em pesquisa realizada em ambiente virtual. O participante obteve acesso ao questionário após aceitação do Termo de Consentimento Livre e Esclarecido (TCLE)^(19)^.

Para a coleta de dados dos profissionais da APS, utilizou-se o instrumento validado EPI-APS COVID-19^(20)^, composto por 31 itens distribuídos em oito domínios: 1) Gorro ou touca descartável; 2) Luvas; 3) Comportamento de segurança; 4) Máscara N95; 5) Higienização das mãos; 6) Avental ou capote descartável; 7) Máscara cirúrgica descartável; 8) Óculos ou máscara de proteção individual. A versão adaptada do instrumento foi usada com os profissionais vinculados aos Programas de Residência em Saúde, com a substituição do termo “serviço da/de APS” por “serviço de saúde” em nove questões e exclusão do termo “APS” em uma questão^(21)^. A equipe de pesquisadores elaborou questões para caracterizar o participante quanto aos aspectos sociodemográficos, de formação profissional e do local de trabalho.

As variáveis dependentes do estudo foram o uso adequado dos EPIs e a adesão. As variáveis independentes foram ser profissional da APS ou vinculados a Programas de Residência em Saúde, idade, a realização ou não de atividades de capacitação voltadas para uso de EPI e as modalidades de realização de atividades de capacitação.

### Análise dos resultados e estatística

Os dados coletados na plataforma KoboToolbox foram exportados para planilha do programa Microsoft Office Excel visando à avaliação de consistência e organização do banco. Posteriormente, para realização das análises estatísticas, foram exportados para o software estatístico Statistical Package for the Social Sciences (SPSS), versão 21.0. Primeiramente foi realizado o teste de normalidade Kolmogorov-Smirnov. Utilizou-se análise estatística descritiva (frequência absoluta e relativa, média e desvio-padrão) para caracterização da amostra. Para análise da adesão ao uso de EPI pelos trabalhadores de saúde, empregaram-se medidas de tendência central (média, mediana) e medidas de dispersão (desvio-padrão e intervalo interquartil).

Para a mensurar o uso adequado do EPI, considerou-se que o profissional faz uso adequado quando alcança a totalidade de pontos avaliados em cada domínio (os itens relativos à falta do EPI não foram utilizados para tal análise). Já a adesão ao uso do EPI foi medida por meio do cálculo individual (número de domínios que apresentou uso adequado / número total de domínios respondidos × 100). A pontuação adotada para adesão ao uso de EPI foi maior e/ou igual a 75% conforme outros estudos encontrados na literatura^(22,23)^. Assim, a adesão determina o grau em que os trabalhadores da APS e os profissionais vinculados aos Programas de Residência em Saúde seguem o uso adequado dos EPIs para protegerem-se contra a contaminação pela COVID-19.

Foram utilizados os testes qui-quadrado ou exato de Fisher para analisar a associação entre variáveis dependentes e independentes, adotando-se o valor de significância *p* ≤ 0,05. As prevalências relacionadas à realização de atividades de capacitação com o uso adequado dos EPIs foram estimadas com intervalo de confiança de 95% (IC 95%). A relação entre a adesão e a realização de atividades de capacitação sobre EPI pelos trabalhadores de saúde e residentes foi analisada por meio do teste de Mann-Whitney.

## RESULTADOS

Participaram do estudo 557 trabalhadores da saúde, sendo 455 (81,7%) da APS e 102 (18,3%) vinculados a Programas de Residência em Saúde. A média de idade encontrada foi de 37,3 anos (desvio-padrão - DP±8,9) para trabalhadores da APS e 28,3 anos (DP±6,7) para vinculados a Programas de Residência. Do total dos participantes, a faixa etária predominante foi de 25 a 39 anos (344; 61,8%), gênero mulher cis (449; 80,6%), com companheiro(a) (279; 50,1%) e residentes na Região Sudeste do Brasil (376; 67,5%).

Quanto aos dados profissionais, 443 (79,5%) têm formação em nível superior, dos quais a maioria (281; 63,4%) relatou especialização na área de saúde. A categoria profissional predominante foi a dos enfermeiros, com 235 participantes (42,2%). A média do tempo de atuação na APS foi de 9,6 anos (DP±7,7); e, nos Programas de Residência, de 13,8 meses (DP±9,5).

Quando questionados sobre terem domínio de conhecimentos e técnicas referentes aos EPIs recomendados para os serviços de saúde onde atuam, 278 (49,9%) relataram concordar parcialmente com a afirmação. A maior parte dos participantes (454; 81,5%) concordou totalmente com a necessidade de realizar atividades de capacitação relacionadas ao uso de EPI.

A caracterização ligada às atividades de capacitação sobre EPI está descrita na Tabela 1. Observou-se que 305 (54,8%) participantes relataram ter realizado atividades de capacitação no período da pandemia.

**Tabela 1:** Caracterização de atividades de capacitação sobre equipamentos de proteção individual por trabalhadores da Atenção Primária à Saúde e por profissionais vinculados a Programas de Residência em Saúde. Brasil, 2021 (N = 557)

Variáveis	Vínculo profissional do participante							
	APS n (%)	Residência n (%)
Realização de atividades de capacitação	Sim	248 (54,5)	57 (55,9)
	Não	207 (45,5)	45 (44,1)
Modalidade			
On-line / Ensino à distância	Sim	188 (75,8)	48 (84,2)
	Não	60 (24,2)	9 (15,8)
Presencial	Sim	76 (30,6)	21 (36,8)
	Não	172 (69,4)	36 (63,2)
Semipresencial	Sim	10 (4,0)	1 (1,8)
	Não	238 (96,0)	56 (98,2)
Carga horária*			
1 a 4 horas		86 (34,7)	16 (28,1)
5 a 10 horas		52 (21,0)	9 (15,8)
11 a 30 horas		51 (20,6)	21 (36,8)
Acima de 31 horas		58 (23,4)	11 (19,3)
Não informado		1 (0,4)	0 (0,0)

* Categorização da carga horária conforme percentis.

Houve associação entre a realização de atividades de capacitação sobre EPI e o uso adequado de luvas (p < 0,001), avental/capote (p = 0,009) e óculos/proteção facial (p = 0,002) entre os trabalhadores da APS. A prevalência de uso adequado de EPI entre os trabalhadores da APS que realizaram atividades de capacitação foi 1,56 vez maior para o uso de luvas, 1,54 vez maior para o uso de avental/capote e 1,37 vez maior para o uso de óculos/protetor facial quando comparados aos trabalhadores que não realizaram atividades de capacitação (Tabela 2).

**Tabela 2:** Associação entre a realização de atividades de capacitação e o uso adequado de equipamento de proteção individual, comportamento de segurança e higiene das mãos entre trabalhadores da Atenção Primária à Saúde. Brasil, 2021 (n = 455)

Atividades de capacitação	Uso adequado de EPI n (%)							
	**Gorro**		**Luvas**		**Avental/Capote**		**Óculos/Protetor facial**	
	**Sim**	**Não**	**Sim**	**Não**	**Sim**	**Não**	**Sim**	**Não**
Sim	26 (15,2)	145 (84,8)	83 (48,5)	88 (51,5)	74 (48,7)	78 (51,3)	114 (71,2)	46 (28,8)
Não	13 (10,1)	116 (89,9)	40 (31,0)	89 (69,0)	29 (31,5)	63 (68,5)	52 (52,0)	48 (48,0)
Valor de*p*	0,191*		<0,001*		0,009*		0,002*	
RP (IC 95%)	1,50 (0,807-2,820)		1,56 (1,159-2,113)		1,54 (1,096-2,175)		1,37 (1,108-1,695)	
**Atividades de capacitação**	**Uso adequado de EPI n (%)**				**Comportamento de segurança n (%)**		**Higienização das mãos n (%)**	
	**Máscara cirúrgica**		**Máscara N95**					
	**Sim**	**Não**	**Sim**	**Não**	**Sim**	**Não**	**Sim**	**Não**
Sim	52 (24,8)	158 (75,2)	79 (59,4)	54 (40,6)	35 (14,1)	213 (85,9)	132 (53,2)	116 (46,8)
Não	48 (27,4)	127 (72,6)	40 (46,0)	47 (54,0)	20 (9,7)	187 (90,3)	92 (44,4)	115 (55,6)
Valor de*p*	0,552*		0,051*		0,147*		0,062*	
RP (IC 95%)	0,90 (0,644-1,265)		1,29 (0,989-1,688)		1,46 (0,871-2,451)		1,19 (0,989-1,451)	

EPI – equipamento de proteção individual; n – frequência absoluta; RP – razão de prevalência; IC 95% – intervalo de confiança de 95%; * teste qui-quadrado.

Na análise da associação entre a realização de atividades de capacitação e o uso adequado de EPI pelos profissionais vinculados aos Programas de Residência em Saúde, foram obtidos valores estatisticamente significantes para o uso adequado de máscara cirúrgica (p=0,028). Esse uso foi 1,42 vez maior entre os residentes que realizaram atividades de capacitação quando comparados àqueles que não as fizeram (Tabela 3).

**Tabela 3:** Associação entre a realização de atividades de capacitação e o uso adequado de equipamento de proteção individual, comportamento de segurança e higiene das mãos por profissionais vinculados a Programas de Residência em Saúde. Brasil, 2021 (n = 102)

**Atividades de capacitação**	**Uso adequado de EPI n (%)**							
	**Gorro**		**Luvas**		**Avental/Capote**		**Óculos/Protetor facial**	
	**Sim**	**Não**	**Sim**	**Não**	**Sim**	**Não**	**Sim**	**Não**
Sim	9 (23,7)	29 (76,3)	0 (0,0)	43 (100,0)	9 (26,5)	25 (73,5)	22 (57,9)	16 (42,1)
Não	4 (17,4)	19 (82,6)	3 (11,1)	24 (88,9)	8 (40,0)	12 (60,0)	6 (42,9)	8 (57,1)
Valor de*p*	0,749€		0,053€		0,301*		0,335*	
RP (IC 95%)	1,36 (0,473-3,922)		------		0,66 (0,305-1,438)		1,351 (0,696-2,621)	
**Atividades de capacitação**	**Uso adequado de EPI n (%)**				**Comportamento de segurança n (%)**		**Higienização das mãos n (%)**	
	**Máscara cirúrgica**		**Máscara N95**					
	**Sim**	**Não**	**Sim**	**Não**	**Sim**	**Não**	**Sim**	**Não**
Sim	36 (75,0)	12 (25,0)	19 (52,8)	17 (47,2)	1 (1,7)	59 (98,3)	21 (35,0)	39 (65,0)
Não	21 (52,5)	19 (47,5)	8 (44,4)	10 (55,6)	0 (0,0)	42 (100,0)	13 (31,0)	29 (69,0)
Valor de*p*	0,028*		0,564*		1,000€		0,670*	
RP (IC 95%)	1,42 (1,020-2,001)		1,18 (0,650-2,168)		------		1,13 (0,641-1,996)	

EPI – equipamento de proteção individual; n – frequência absoluta; RP – razão de prevalência; IC 95% – intervalo de confiança de 95%; * teste qui-quadrado; ^€^ teste exato de Fisher.

Por um lado, quando avaliado se o vínculo profissional está associado ao uso adequado de EPI, comportamento de segurança e higiene das mãos (Tabela 4), verificou-se uma diferença estatisticamente significante em que o grupo APS apresentou prevalência 3,28 vezes maior de uso adequado de luvas (p < 0,001) e 1,35 vez maior de higienização das mãos (p = 0,018). Por outro lado, o uso de máscara cirúrgica foi 47% menor (p < 0,001) entre os profissionais da APS quando comparados aos profissionais dos programas de residência.

**Tabela 4:** Associação entre o vínculo profissional e o uso adequado de equipamento de proteção individual, comportamento de segurança e higiene das mãos. Brasil, 2021 (N = 557)

**Vínculo profissional**	**Uso adequado de EPI n (%)**							
	**Gorro**		**Luvas**		**Avental/Capote**		**Óculos/Protetor facial**	
	**Sim**	**Não**	**Sim**	**Não**	**Sim**	**Não**	**Sim**	**Não**
APS	39 (13,0)	261 (87,0)	123 (41)	177 (59,0)	10 (42,2)	141 (57,8)	166 (63,8)	94 (36,2)
Residência	7 (11,5)	54 (88,5)	8 (12,5)	56 (87,5)	21 (43,8)	27 (56,2)	28 (57,1)	21 (42,9)
Valor de*p*	0,745*		<0,001*		0,844*		0,373*	
RP (IC 95%)	1,13 (0,532-2,413		3,28 (1,691-6,360)		0,96 (0,678-1,373)		1,11 (0,862-1,448)	
**Vínculo profissional**	**Uso adequado de EPI n (%)**				**Comportamento de segurança n (%)**		**Higienização das mãos n (%)**	
	**Máscara cirúrgica**				**Máscara N95**			
	**Sim**	**Não**	**Sim**	**Não**	**Sim**	**Não**	**Sim**	**Não**
APS	100 (26,0)	285 (74,0)	119 (54,1)	101 (45,9)	55 (12,1)	400 (87,9)	224 (49,2)	231 (50,8)
Residência	43 (49,4)	44 (50,6)	31 (49,2)	32 (50,8)	0 (0,0)	102 (100,0)	37 (36,3)	65 (63,7)
Valor de*p*	<0,001*		0,493*		<0,001€		0,018*	
RP (IC 95%)	0,526 (0,401-0,689)		1,09 (0,832-1,453)		------		1,35 (1,032-1,784)	

EPI – equipamento de proteção individual; APS - Atenção Primária à Saúde; n – frequência absoluta; RP – razão de prevalência; IC 95% – intervalo de confiança de 95%; * teste qui-quadrado; ^€^ teste exato de Fisher.

Dentre os aspectos dificultadores do uso adequado de EPI no serviço de saúde (dados não mostrados em tabela), os participantes mencionaram a ausência de atividades de capacitação (291; 52,2%), a falta de conhecimento (227; 40,8%), a ausência de infraestrutura (224; 40,2%) e a falta do EPI (310; 55,7%).

Quanto à adesão ao uso adequado de EPI pelos trabalhadores da APS (Tabela 5), foi observado que 17 (3,7%) apresentaram 75% de adesão, 14 (3%) apresentaram valores de 76% a 99% de adesão e apenas 1 (0,2%) apresentou 100% de adesão ao uso de EPI. No grupo residentes, nenhum participante apresentou adesão ao uso de EPI igual ou superior a 75% (Tabela 5).

**Tabela 5:** Adesão ao uso adequado de equipamento de proteção individual por trabalhadores da Atenção Primária à Saúde e por profissionais vinculados a Programas de Residência em Saúde. Brasil, 2021 (N = 557)

Vínculo profissional	Mínimo	1º Quartil	Média (DP)	Mediana	3º Quartil	Máximo
APS (%)	0,0	12,5	33,3 (±25,0)	33,3	50,0	100,0
Residentes (%)	0,0	16,2	28,4 (±19,0)	29,0	43,0	67,0

*APS - Atenção Primária à Saúde; DP – desvio-padrão.*

Na análise de relação entre a adesão e a realização de atividades de capacitação sobre EPI pelos trabalhadores de saúde, foi observado que tais atividades de capacitação têm efeito sobre a adesão ao uso de EPI (p<0,001).

## DISCUSSÃO

Este estudo avaliou a associação da realização de atividades de capacitação com a adesão e o uso de EPI pelos trabalhadores de saúde da APS e por profissionais vinculados a Programas de Residência atuantes em serviços de APS. Os achados mostraram que a realização de atividades de capacitação está associada ao uso adequado de luvas, avental/capote e óculos/proteção facial entre trabalhadores da APS; e máscara cirúrgica entre residentes. Essas atividades tiveram efeito na adesão ao uso de EPI em ambos os grupos.

Atividades de capacitação foram associadas ao uso adequado de EPI em outras investigações^(1,24)^. Sabe-se que o conhecimento e compreensão dos princípios de biossegurança bem como o manuseio correto dos EPIs durante seu uso, paramentação e desparamentação levam à segurança do paciente e do profissional e, consequentemente, à minimização da contaminação por COVID-19^(7,8,23)^. Para isso, é necessário adotar protocolos de controle de infecções e disponibilizar EPIs como máscaras, aventais, óculos, protetores faciais e luvas^(5)^.

Todavia, os participantes deste estudo apresentaram baixa adesão ao uso de EPI, assim como em outras investigações^(9,25,26)^. Esse fato sugere que há uma desvalorização dos riscos existentes no ambiente laboral por parte dos profissionais^(27)^. A adesão aos EPIs como medida preventiva à COVID-19 encontra-se ligada a diversos fatores: i) organizacionais, como clima de segurança, diretrizes e disponibilidade de programas de treinamentos^(13,22,28)^; ii) ambientais, como o próprio ambiente físico e a disponibilidade de EPI^(13,22,29)^ e iii) fatores individuais, como conhecimento^(11)^ e atitudes positivas quanto à escolha e ao uso do EPI e maior tempo de experiência clínica^(22,29)^.

No tocante ao uso adequado de EPI pelos trabalhadores da APS, destaca-se que o item “óculos/protetor facial” foi o que apresentou o maior percentual. Tal evidência pode ser justificada pela importância desse EPI para a proteção dos trabalhadores em atividades com exposição a secreções corporais^(4,30)^. Em consonância, pesquisa on-line realizada no Catar por meio de um questionário autoaplicável com a participação de 757 profissionais da APS mostrou que o uso de óculos/protetor facial foi referido por 55,4% dos trabalhadores durante os atendimentos a casos suspeitos ou confirmados de COVID-19^(30)^. Já no Brasil, estudo realizado com 106 profissionais de saúde bucal mostrou o uso adequado das medidas de precauções para aerossóis, especialmente o protetor facial (75,5%), a máscara N95 (62,3%) e o avental/capote (53,8%)^(31)^.

Com relação a máscara cirúrgica, o uso adequado em atendimentos a casos suspeitos ou confirmados de COVID-19 apresentou um percentual de 90,6% em trabalhadores da APS em estudo prévio^(30)^. Porém, no presente estudo, seu maior uso se deu no grupo de profissionais residentes, resultado que pode ser atribuído ao fato de os residentes estarem em formação no serviço, sendo, por isso, frequentemente avaliados pela equipe e pelos preceptores quanto aos comportamentos adotados para assegurar a proteção contra a contaminação pelo vírus causador da COVID-19.

Investigações realizadas no período da pandemia de COVID-19 mostraram que as luvas estavam entre os EPIs mais utilizados^(30,31,32)^. No presente estudo, os trabalhadores da APS apresentaram a prevalência de 3,28 vezes maior de uso adequado de luvas do que os profissionais residentes. O gorro foi o EPI do qual os participantes tiveram maior dificuldade em realizar o uso adequado, corroborando pesquisa realizada no período pré-pandemia com profissionais de enfermagem de serviços hospitalares^(33)^. No entanto, outro estudo mostrou que profissionais de saúde bucal referiram o gorro como o EPI mais utilizado durante a pandemia^(31)^.

Na presente pesquisa, os principais dificultadores relatados pelos trabalhadores da saúde para o uso adequado do EPI foram a ausência de atividades de capacitação e a falta de conhecimento sobre medidas de controle e prevenção. No entanto, esse resultado não se restringe apenas à COVID-19. Estudo recente realizado em serviços de APS e de urgência do Colorado evidenciou que apenas 23% dos profissionais de saúde expostos à *monkeypox* faziam uso de todos os EPIs recomendados (máscara, luvas, aventais, óculos ou *face shield*)^(34)^. Essa baixa adesão pode ser justificada pela falta de conhecimento das manifestações clínicas da doença, ocorrência de transmissão comunitária e recomendações sobre EPI^(35)^. Dessa forma, já que os profissionais de saúde atuantes na linha de frente de combate à covid têm maior probabilidade de contato com o vírus, é preciso proporcionar-lhes atividades de capacitação para que adotem medidas conscientes e seguras em seu ambiente laboral^(34)^.

O comportamento de segurança, que é um domínio avaliado pelo instrumento de coleta de dados utilizado na presente pesquisa, apresentou o pior resultado em ambas as categorias profissionais participantes. Esse achado corrobora um estudo brasileiro no qual se demonstrou que nem todos os cuidados de biossegurança são realizados no cotidiano de trabalho^(31)^. Além disso, associado ao uso adequado dos EPIs para o desenvolvimento seguro das atividades laborais dos profissionais, é necessária a adesão a práticas como higienização das mãos^(25)^ e descontaminação do ambiente^(36)^.

Destaca-se que a adequada higienização das mãos foi referida por 49,2% dos trabalhadores da APS e 36,3% dos residentes. Já na Etiópia, 56,7% dos trabalhadores apresentaram boas práticas de higienização das mãos durante a pandemia de COVID-19^(25)^. Entretanto, estudo realizado com trabalhadores da APS do Brasil antes da pandemia de COVID-19 evidenciou que, dentre os pontos dificultadores da não adoção de medidas-padrão de precaução, estão a percepção de que os riscos para infecção na APS são baixos quando comparados ao ambiente hospitalar e de que há fragilidades na oferta de capacitações^(37)^.

Quanto às atividades de capacitação, diante do cenário pandêmico, da importância de medidas preventivas como o distanciamento social e da necessidade de constante formação profissional, os trabalhadores da APS e os residentes realizaram atividades de capacitação na modalidade on-line/ensino à distância sobre medidas preventivas de qualidade nos serviços onde atuam. As atividades de capacitação foram oferecidas no formato on-line/ teórico com carga horária reduzida devido à urgência de ofertar aos profissionais de saúde informações relevantes e altamente demandadas para combater a COVID-19^(16,17)^.

A literatura evidenciou que, em vários países (como Austrália^(15)^, Brasil^(26)^, China^(38)^ e Etiópia^(39)^), os profissionais da saúde receberam capacitações insuficientes para enfrentar a COVID-19. Nesse contexto, destaca-se a importância da atuação das autoridades^(40)^ na oferta de capacitações on-line e gratuitas para os profissionais atuantes na pandemia visando ao conhecimento científico necessário para exercerem suas atividades com toda a segurança possível^(19,26)^. Os pesquisadores da presente pesquisa, como forma de translação do conhecimento científico, elaboraram o curso “Biossegurança: boas práticas para a atuação diante da COVID-19”, ofertado a todos os participantes do estudo, de maneira virtual e gratuita, e estruturado em três módulos, com carga horária de 15 horas e certificação^(19)^.

Assim, o investimento em recursos e atividades educacionais para esses trabalhadores representa um grande impacto na qualidade da assistência, pois atua na prevenção das IRAS, como a COVID-19^(25)^. Nesse sentido, profissionais qualificados proporcionam maior segurança às suas equipes e, consequentemente, maior encorajamento para fazer frente à pandemia^(7,15)^.

### Limitações do estudo

Uma limitação está no tipo do estudo, transversal, que deve ser interpretado levando em consideração suas particularidades. Além disso, a pesquisa foi realizada em ambiente virtual, o qual favorece o viés de seleção^(41)^, refletindo uma amostra de participantes que foram sensibilizados por meio das estratégias usadas para divulgar o estudo e que, de forma esclarecida e voluntária, aceitaram participar da pesquisa.

### Contribuições para a Área

Este estudo revela dados preocupantes sobre o uso adequado dos EPIs e sua adesão durante a pandemia de COVID-19, em uma amostra composta por trabalhadores da saúde e residentes em serviços da APS brasileira (42,2% com curso superior em Enfermagem). Esses resultados sugerem possíveis lacunas tanto na formação acadêmica quanto na formação em serviço no que diz respeito às medidas de biossegurança. Sugere-se a inclusão do tema “biossegurança” de forma transversal durante o processo formativo visando à consolidação dos conhecimentos para aplicação consistente na prática. O objetivo disso é garantir a segurança dos trabalhadores e usuários e qualificar a assistência prestada para prevenir a transmissão das IRAS no ambiente de trabalho, não apenas no contexto da COVID-19, mas também de outras doenças infecciosas.

## CONCLUSÕES

Conclui-se que a realização de atividades de capacitação associa-se com a adesão ao uso de EPI e com o uso adequado de máscara cirúrgica para profissionais residentes no enfrentamento da pandemia de COVID-19; e, no caso dos trabalhadores da APS, associa-se com o uso de luvas, avental/capote e óculos/protetor facial. Os profissionais residentes apresentaram adesão abaixo do valor de referência utilizado neste estudo, enquanto apenas 6,9% dos profissionais da APS atingiram valores iguais ou superiores 75% de adesão.

Sugere-se a realização de estudos futuros para identificar os fatores facilitadores e dificultadores da adesão e uso adequado de EPI nos serviços da APS, inclusive no atual período pós-pandemia.
